# Supplementary material for: The relative abundance of languages: Neutral and non-neutral dynamics
Source: PLoS One. 2021 Dec 29;16(12):e0259162. doi: 10.1371/journal.pone.0259162 (PMC8716027; doi:10.1371/journal.pone.0259162)
Supplement: S1 Appendix — Here we present results for all the countries analyses, see S1 Fig and S1-S3 Tables. (DOCX) [file pone.0259162.s001.docx]

The relative abundance of languages:

neutral and non-neutral dynamics

Luís Borda-de-Água and Stephen P. Hubbell

**SUPPORTING INFORMATION**

**S1 Additional information on the 46 countries analyzed.**

Here we present results for all the countries analyses, see Fig. S1 and Tables S1, S2 and S3.

**Table S1. List of all the countries analyzed, their number of languages, number of individuals, *J*, and the maximum likelihoods of *θ*, *ν, P_s_*. and *J_s_= P_s_ *J*.** Countries with name in italic correspond to poor fitting, as seen from the rank abundance plots (see Fig. S1).

| **COUNTRY** | **Number of Languages** | ***J* (individuals)** | ***θ*** | *ν* | ***P_S_*** | ***J_S_*** |
| --- | --- | --- | --- | --- | --- | --- |
| *Somalia* | 10 | 8616834 | 1.70 | 9.86E-08 | 2.30E-03 | 19819 |
| Malawi | 12 | 10298480 | 2.40 | 1.16E-07 | 4.40E-03 | 45313 |
| Zimbabwe | 15 | 15776670 | 2.60 | 8.24E-08 | 1.90E-03 | 29976 |
| Niger | 21 | 9230862 | 3.40 | 1.84E-07 | 8.70E-04 | 8032 |
| *Sierra Leone* | 21 | 4738750 | 2.40 | 2.53E-07 | 1.10E-04 | 521 |
| Namibia | 23 | 1862707 | 4.60 | 1.23E-06 | 2.20E-03 | 4098 |
| *Botswana* | 27 | 1435017 | 5.00 | 1.74E-06 | 1.39E-03 | 1990 |
| Guinea | 27 | 6640506 | 4.20 | 3.16E-07 | 6.00E-04 | 3984 |
| Liberia | 28 | 2430017 | 10.20 | 2.10E-06 | 1.19E-02 | 28917 |
| *Senegal* | 34 | 9370320 | 5.50 | 2.93E-07 | 6.00E-04 | 5622 |
| Zambia | 37 | 10673260 | 7.90 | 3.70E-07 | 1.90E-03 | 20279 |
| Gabon | 38 | 609423 | 11.90 | 9.76E-06 | 6.00E-03 | 3656 |
| Angola | 38 | 10978412 | 5.80 | 2.64E-07 | 3.70E-04 | 4062 |
| Togo | 38 | 3978381 | 8.00 | 1.00E-06 | 1.50E-03 | 5968 |
| Uganda | 40 | 17004328 | 11.00 | 3.23E-07 | 4.00E-03 | 68017 |
| Mozambique | 41 | 16210221 | 8.10 | 2.50E-07 | 1.20E-03 | 19452 |
| Mali | 46 | 9010979 | 8.90 | 4.94E-07 | 1.00E-03 | 9011 |
| *Benin* | 53 | 6449442 | 17.00 | 1.32E-06 | 4.60E-03 | 29667 |
| Congo | 57 | 3366116 | 12.00 | 1.78E-06 | 1.10E-03 | 3703 |
| Burkina Faso | 64 | 11018638 | 10.00 | 4.54E-07 | 2.20E-04 | 2424 |
| Cent. Afr. Republic | 67 | 3298745 | 16.00 | 2.42E-06 | 1.40E-03 | 4618 |
| Ghana | 73 | 20124060 | 11.00 | 2.73E-07 | 2.20E-04 | 4427 |
| Côte d’Ivoire | 77 | 9150469 | 15.00 | 8.20E-07 | 5.50E-04 | 5033 |
| Tanzania | 113 | 25687658 | 28.00 | 5.45E-07 | 1.10E-03 | 28256 |
| Chad | 123 | 5809406 | 21.00 | 1.81E-06 | 2.30E-04 | 1336 |
| Dem. Rep. Congo | 202 | 38399510 | 35.00 | 4.56E-07 | 1.40E-04 | 5376 |
| Cameroon | 268 | 9637152 | 56.00 | 2.90E-06 | 2.30E-04 | 2216 |
| Guyana | 11 | 676904 | 0.90 | 6.65E-07 | 6.00E-06 | 4.06 |
| Panama | 12 | 2100000 | 0.80 | 1.90E-07 | 1.20E-06 | 2.52 |
| Suriname | 15 | 628827 | 1.40 | 1.11E-06 | 2.40E-05 | 15.1 |
| *Colombia* | 76 | 34571380 | 7.00 | 1.01E-07 | 4.10E-06 | 141 |
| Oman | 12 | 1470063 | 1.30 | 4.42E-07 | 1.36E-04 | 200 |
| *Cambodia* | 19 | 12443935 | 1.70 | 6.83E-08 | 1.60E-05 | 199 |
| *Bangladesh* | 30 | 122662860 | 2.70 | 1.10E-08 | 8.50E-06 | 1043 |
| *Thailand* | 62 | 53529352 | 6.00 | 5.60E-08 | 5.60E-06 | 300 |
| Laos | 80 | 5300189 | 12.00 | 1.13E-06 | 1.90E-04 | 1007 |
| *Vietnam* | 92 | 75273638 | 8.00 | 5.31E-08 | 3.30E-06 | 248 |
| *Myanmar* | 98 | 46606310 | 14.00 | 1.50E-07 | 1.00E-04 | 4661 |
| *Nepal* | 117 | 22813243 | 16.00 | 3.51E-07 | 5.20E-05 | 1186 |
| *Malaysia* | 125 | 15566135 | 15.00 | 4.82E-07 | 2.30E-05 | 358 |
| *Philippines* | 168 | 70626954 | 20.00 | 1.42E-07 | 2.00E-05 | 1412 |
| *India* | 397 | 945679579 | 38.00 | 2.01E-08 | 1.30E-06 | 1229 |
| *Indonesia* | 724 | 218610076 | 70.00 | 1.60E-07 | 6.90E-07 | 151 |
| Solomon Islands | 69 | 353992 | 29.00 | 4.10E-05 | 6.70E-03 | 2372 |
| Vanuatu | 109 | 117494 | 30.00 | 1.28E-04 | 1.45E-03 | 170 |
| Papua New Guinea | 819 | 3665383 | 193.00 | 2.63E-05 | 1.20E-04 | 440 |

**Table S2. List of all the countries analyzed, the maximum likelihoods of *θ*, and *P_s_* and their corresponding 95% confidence intervals.** Countries with name in italic correspond to poor fitting, as seen from the rank abundance plots (see Fig. S1).

| **COUNTRY** | ***θ***  **-95%CI** | ***θ*** | ***θ***  **+95%CI** | ***P_S_***  **-95%CI** | ***P_S_*** | ***P_S_***  **+95%CI** |
| --- | --- | --- | --- | --- | --- | --- |
| *Somalia* | 0.30 | 1.70 | 7.20 | 2.70E-04 | 2.30E-03 | 6.27E-03 |
| Malawi | 0.80 | 2.40 | 9.90 | 1.90E-03 | 4.40E-03 | 3.98E-02 |
| Zimbabwe | 0.90 | 2.60 | 10.00 | 7.00E-04 | 1.90E-03 | 1.53E-02 |
| Niger | 1.10 | 3.40 | 9.80 | 3.70E-04 | 8.70E-04 | 2.57E-03 |
| *Sierra Leone* | 1.10 | 2.40 | 10.40 | 1.20E-04 | 1.10E-04 | 7.34E-03 |
| Namibia | 1.80 | 4.60 | 12.50 | 9.60E-04 | 2.20E-03 | 8.74E-03 |
| *Botswana* | 1.80 | 5.00 | 12.30 | 5.40E-04 | 1.39E-03 | 3.31E-03 |
| Guinea | 1.50 | 4.20 | 11.20 | 2.80E-04 | 6.00E-04 | 1.51E-03 |
| Liberia | 5.10 | 10.20 | 36.20 | 4.00E-03 | 1.19E-02 | 6.90E-02 |
| *Senegal* | 2.20 | 5.50 | 13.30 | 2.30E-04 | 6.00E-04 | 1.29E-03 |
| Zambia | 3.90 | 7.90 | 19.00 | 8.00E-04 | 1.90E-03 | 6.70E-03 |
| Gabon | 5.50 | 11.90 | 24.50 | 3.10E-03 | 6.00E-03 | 1.36E-02 |
| Angola | 2.50 | 5.80 | 15.00 | 1.00E-04 | 3.70E-04 | 2.00E-03 |
| Togo | 3.50 | 8.00 | 20.00 | 8.00E-04 | 1.50E-03 | 8.80E-03 |
| Uganda | 5.50 | 11.00 | 26.00 | 1.70E-03 | 4.00E-03 | 1.54E-02 |
| Mozambique | 3.50 | 8.10 | 20.00 | 4.00E-04 | 1.20E-03 | 8.50E-03 |
| Mali | 4.00 | 8.90 | 19.50 | 3.00E-04 | 1.00E-03 | 3.20E-03 |
| *Benin* | 9.50 | 17.00 | 34.00 | 2.90E-03 | 4.60E-03 | 1.06E-02 |
| Congo | 6.00 | 12.00 | 22.50 | 7.40E-04 | 1.10E-03 | 2.21E-03 |
| Burkina Faso | 5.00 | 10.00 | 21.00 | 1.20E-04 | 2.20E-04 | 1.06E-03 |
| Cent. Afr. Republic | 8.50 | 16.00 | 29.50 | 8.50E-04 | 1.40E-03 | 3.64E-03 |
| Ghana | 6.00 | 11.00 | 22.00 | 1.30E-04 | 2.20E-04 | 5.21E-04 |
| Côte d’Ivoire | 8.00 | 15.00 | 26.50 | 2.60E-04 | 5.50E-04 | 1.28E-03 |
| Tanzania | 17.00 | 28.00 | 43.00 | 5.20E-04 | 1.10E-03 | 1.57E-03 |
| Chad | 13.00 | 21.00 | 34.50 | 1.53E-04 | 2.30E-04 | 4.03E-04 |
| Dem. Rep. Congo | 24.50 | 35.00 | 52.00 | 1.03E-04 | 1.40E-04 | 2.38E-04 |
| Cameroon | 40.00 | 56.00 | 75.00 | 1.48E-04 | 2.30E-04 | 3.08E-04 |
| Guyana | 0.26 | 0.90 | 6.03 | 7.00E-06 | 6.00E-06 | 4.31E-04 |
| Panama | 0.28 | 0.80 | 6.12 | 1.00E-06 | 1.20E-06 | 4.23E-04 |
| Suriname | 0.48 | 1.40 | 7.04 | 1.90E-05 | 2.40E-05 | 1.53E-03 |
| *Colombia* | 3.23 | 7.00 | 14.65 | 1.30E-06 | 4.10E-06 | 8.30E-06 |
| Oman | 0.40 | 1.30 | 6.88 | 5.00E-05 | 1.36E-04 | 2.22E-03 |
| *Cambodia* | 0.54 | 1.70 | 7.28 | 6.00E-06 | 1.60E-05 | 1.75E-04 |
| *Bangladesh* | 0.80 | 2.70 | 8.60 | 4.20E-06 | 8.50E-06 | 2.59E-05 |
| *Thailand* | 2.60 | 6.00 | 13.60 | 3.10E-06 | 5.60E-06 | 2.27E-05 |
| Laos | 6.50 | 12.00 | 23.00 | 9.00E-05 | 1.90E-04 | 4.20E-04 |
| *Vietnam* | 4.10 | 8.00 | 16.40 | 1.50E-06 | 3.30E-06 | 5.40E-06 |
| *Myanmar* | 7.90 | 14.00 | 24.70 | 5.10E-05 | 1.00E-04 | 1.91E-04 |
| *Nepal* | 8.80 | 16.00 | 26.00 | 2.10E-05 | 5.20E-05 | 8.20E-05 |
| *Malaysia* | 8.60 | 15.00 | 25.40 | 1.20E-05 | 2.30E-05 | 4.20E-05 |
| *Philippines* | 12.70 | 20.00 | 32.40 | 9.00E-06 | 2.00E-05 | 4.10E-05 |
| *India* | 27.00 | 38.00 | 54.00 | 8.25E-07 | 1.30E-06 | 2.15E-06 |
| *Indonesia* | 54.00 | 70.00 | 90.00 | 4.80E-07 | 6.90E-07 | 1.00E-06 |
| Solomon Islands | 15.00 | 29.00 | 48.00 | 2.80E-03 | 6.70E-03 | 1.09E-02 |
| Vanuatu | 20.00 | 30.00 | 49.00 | 1.17E-03 | 1.45E-03 | 2.79E-03 |
| Papua New Guinea | 160.00 | 193.00 | 226.00 | 9.40E-05 | 1.20E-04 | 1.40E-04 |

**Table S3. Corrected Akaike Information Criterion values (AICc) for the Allen-Savage (AS) and lognormal (logn) distributions, their weights (*w*) (Burnham and Anderson 2010) and their ratio, *w*_AS_/*w*_logn_.** The ratios, *w*_AS_/*w*_logn_, in red correspond to those cases where there is substantial support to the lognormal distribution, and the ones in green to substantial support to the Allen-Savage distribution.

| **Country** | **AICc AS** | **AICc logn** | ***w*_AS_** | ***w*_logn_** | ***w*_AS_/*w*_logn_** |
| --- | --- | --- | --- | --- | --- |
| Somalia | 268.5 | 266.7 | 2.953E-01 | 7.047E-01 | 4.190E-01 |
| Malawi | 345.6 | 345.0 | 4.329E-01 | 5.671E-01 | 7.634E-01 |
| Zimbabwe | 432.5 | 431.5 | 3.705E-01 | 6.295E-01 | 5.886E-01 |
| Niger | 548.4 | 550.8 | 7.703E-01 | 2.297E-01 | 3.353E+00 |
| Sierra Leone | 548.9 | 548.5 | 4.477E-01 | 5.523E-01 | 8.106E-01 |
| Namibia | 548.4 | 549.0 | 5.842E-01 | 4.158E-01 | 1.405E+00 |
| Botswana | 589.3 | 583.9 | 6.538E-02 | 9.346E-01 | 6.995E-02 |
| Guinea | 672.4 | 675.1 | 8.006E-01 | 1.994E-01 | 4.015E+00 |
| Liberia | 696.3 | 695.8 | 4.428E-01 | 5.572E-01 | 7.945E-01 |
| Senegal | 851.6 | 852.7 | 6.248E-01 | 3.752E-01 | 1.665E+00 |
| Zambia | 985.4 | 987.2 | 7.171E-01 | 2.829E-01 | 2.535E+00 |
| Gabon | 807.0 | 806.2 | 3.989E-01 | 6.011E-01 | 6.637E-01 |
| Angola | 981.9 | 979.7 | 2.497E-01 | 7.503E-01 | 3.329E-01 |
| Togo | 943.6 | 942.1 | 3.230E-01 | 6.770E-01 | 4.771E-01 |
| Uganda | 1115.8 | 1117.3 | 6.835E-01 | 3.165E-01 | 2.160E+00 |
| Mozambique | 1130.6 | 1131.8 | 6.457E-01 | 3.543E-01 | 1.822E+00 |
| Mali | 1185.4 | 1185.4 | 4.950E-01 | 5.050E-01 | 9.802E-01 |
| Benin | 1332.3 | 1333.0 | 5.915E-01 | 4.085E-01 | 1.448E+00 |
| Congo | 1310.6 | 1307.8 | 2.010E-01 | 7.990E-01 | 2.516E-01 |
| Burkina Faso | 1581.1 | 1572.2 | 1.166E-02 | 9.883E-01 | 1.180E-02 |
| Cent. Afr. Republic | 1567.3 | 1576.2 | 9.885E-01 | 1.154E-02 | 8.563E+01 |
| Ghana | 1855.8 | 1853.7 | 2.592E-01 | 7.408E-01 | 3.499E-01 |
| Côte d’Ivoire | 1899.2 | 1898.4 | 4.110E-01 | 5.890E-01 | 6.977E-01 |
| Tanzania | 2975.4 | 2983.5 | 9.832E-01 | 1.679E-02 | 5.856E+01 |
| Chad | 2783.1 | 2790.8 | 9.790E-01 | 2.104E-02 | 4.653E+01 |
| Dem. Rep. Congo | 5051.9 | 5050.7 | 3.475E-01 | 6.525E-01 | 5.326E-01 |
| Cameroon | 5905.0 | 5918.2 | 9.987E-01 | 1.305E-03 | 7.651E+02 |
| Guyana | 219.6 | 217.3 | 2.460E-01 | 7.540E-01 | 3.263E-01 |
| Panama | 276.6 | 269.8 | 3.137E-02 | 9.686E-01 | 3.239E-02 |
| Suriname | 324.7 | 327.0 | 7.595E-01 | 2.405E-01 | 3.158E+00 |
| Colombia | 1505.3 | 1450.3 | 1.117E-12 | 1.000E+00 | 1.117E-12 |
| Oman | 288.8 | 290.4 | 6.964E-01 | 3.036E-01 | 2.293E+00 |
| Cambodia | 428.7 | 421.5 | 2.686E-02 | 9.731E-01 | 2.760E-02 |
| Bangladesh | 772.2 | 770.8 | 3.340E-01 | 6.660E-01 | 5.016E-01 |
| Thailand | 1532.1 | 1522.0 | 6.181E-03 | 9.938E-01 | 6.220E-03 |
| Laos | 1769.0 | 1759.1 | 7.034E-03 | 9.930E-01 | 7.083E-03 |
| Vietnam | 2170.4 | 2142.5 | 8.777E-07 | 1.000E+00 | 8.777E-07 |
| Myanmar | 2460.4 | 2436.6 | 6.676E-06 | 1.000E+00 | 6.676E-06 |
| Nepal | 2693.1 | 2674.7 | 9.903E-05 | 9.999E-01 | 9.904E-05 |
| Malaysia | 2690.8 | 2664.8 | 2.283E-06 | 1.000E+00 | 2.283E-06 |
| Philippines | 4134.2 | 4094.6 | 2.492E-09 | 1.000E+00 | 2.492E-09 |
| India | 10474.8 | 10408.8 | 4.613E-15 | 1.000E+00 | 4.613E-15 |
| Indonesia | 15709.1 | 15504.8 | 4.163E-45 | 1.000E+00 | 4.163E-45 |
| Solomon Islands | 1320.9 | 1333.4 | 9.980E-01 | 2.005E-03 | 4.977E+02 |
| Vanuatu | 1709.1 | 1722.4 | 9.988E-01 | 1.242E-03 | 8.043E+02 |
| Papua New Guinea | 14800.1 | 14784.7 | 4.526E-04 | 9.995E-01 | 4.528E-04 |

**Fig. S1.** **Language abundance distributions for the countries listed in Tables S1 and S2.** For each country the top plot is the histograms of the language abundance distribution and the fitted Allen-Savage curve, red line, and the best fit lognormal distribution, blue line. The bins are centered in integers numbers, *n*, and have borders at *n*±0.5. The bottom plots are rank abundance distributions. Languages are ranked from the most abundant on the left-hand side of the *x*-axis to the least abundant on the right-hand side. The errors bars correspond to 95% confidence intervals and were obtained for each country by sampling 200 times a number of points equal to the number of languages from a distribution with parameters corresponding to the maximum likelihood estimates. Plots for Somalia, Malawi and Zimbabwe.

**Fig. S1.** **Language abundance distributions for the countries listed in Tables S1 and S2 (continuation).** Plots for Niger, Sierra Leone and Namibia.

**Fig. S1.** **Language abundance distributions for the countries listed in Tables S1 and S2 (continuation).** Plots for Botswana, Guinea and Liberia.

**Fig. S1.** **Language abundance distributions for the countries listed in Tables S1 and S2 (continuation).** Plots for Senegal, Zambia and Gabon.

**Fig. S1.** **Language abundance distributions for the countries listed in Tables S1 and S2 (continuation).** Plots for Angola, Togo and Uganda.

**Fig. S1.** **Language abundance distributions for the countries listed in Tables S1 and S2 (continuation).** Plots for Mozambique, Mali and Benin.

**Fig. S1.** **Language abundance distributions for the countries listed in Tables S1 and S2 (continuation).** Plots for Congo, Burkina Faso and Central African Republic.

**Fig. S1.** **Language abundance distributions for the countries listed in Tables S1 and S2 (continuation).** Plots for Ghana, Côte d’Ivoire and Tanzania.

**Fig. S1.** **Language abundance distributions for the countries listed in Tables S1 and S2 (continuation).** Plots for Chad, Republic Democratic of Congo and Cameroon.

**Fig. S1.** **Language abundance distributions for the countries listed in Tables S1 and S2 (continuation).** Plots for Guyana, Panama and Suriname.

**Fig. S1.** **Language abundance distributions for the countries listed in Tables S1 and S2 (continuation).** Plots for Colombia, Oman and Cambodia.

**Fig. S1.** **Language abundance distributions for the countries listed in Tables S1 and S2 (continuation).** Plots for Bangladesh, Thailand and Laos.

**Fig. S1.** **Language abundance distributions for the countries listed in Tables S1 and S2 (continuation).** Plots for Vietnam, Myanmar and Nepal.

**Fig. S1.** **Language abundance distributions for the countries listed in Tables S1 and S2 (continuation).** Plots for Malaysia, Philippines and India.

**Fig. S1.** **Language abundance distributions for the countries listed in Tables S1 and S2 (continuation).** Plots for Indonesia, Solomon Islands and Vanuatu.

**Fig. S1.** **Language abundance distributions for the countries listed in Tables S1 and S2 (continuation).** Plot for Papua New Guinea.
