# Supplementary material for: The relative abundance of languages: Neutral and non-neutral dynamics
Source: PLoS One. 2021 Dec 29;16(12):e0259162. doi: 10.1371/journal.pone.0259162 (PMC8716027; doi:10.1371/journal.pone.0259162)
Supplement: S2 Appendix — (DOCX) [file pone.0259162.s002.docx]

The relative abundance of languages:

neutral and non-neutral dynamics

Luís Borda-de-Água and Stephen P. Hubbell

**SUPPORTING INFORMATION**

**S2 Population Growth and the Allen-Savage Distribution.**

We consider a community (country) with *L* languages and total number of speakers *J*. Under the Neutral Theory assumptions, *in equilibrium* the number of speakers of a given language, *N_i_*, undergoes random drift (eventually becoming extinct), but the total community size, *J*, and the shape of the language abundance distribution remain the same.

The assumption of a constant number of individuals, *J*, is clearly not true for most human societies in the last centuries, hence, the obvious question is: what is the impact of population growth on the shape of the language abundance distribution? We approach this problem by looking at which transformations of the random variable *N_i_* (or *P_i_*= *N_i_*/*J*) preserve the distribution of the language abundance distributions. In other words, if the distribution of abundances at *t*=0 is *L_i_*_0_ and at time *t*>0 is *L_i_*(*t*), which transformations are such that *L_i_*_0_ and *L_i_*(*t*) are described by the same distribution?

In general, given two random variables, *X* and *Y* with cumulative density functions *F*(*x|****φ***_1_) and *H*(*y|****φ***_2_), where ***φ***_1_ and ***φ***_2_ are vectors of parameters, we know that *U*=*F*(*X|****φ***_1_) and *U*=*H*(*Y|****φ***_2_), where *U* has the uniform distribution in (0,1). If *Y*=*g*(*X*) then, using the previous identities, *y*=*g*(*x*)=*H*^-1^(*F*(*x|****φ***_1_) *|****φ***_2_), where *H*^-1^ is the inverse function of *H*, i.e., the quantile function. If *H*(*y*) has the same form of *F*(*x*), but eventually with different parameters, then the transformation of variables that preserves the shape of the distribution is

*g*(*x*)=*F*^-1^(*F*(*x*|***φ***_1_)|***φ***_2_).

The complexity of the Allen-Savage distribution, Eq. 1, precludes us of calculating its quantile function, and so of obtaining *g*.
 One case where growth preserves the distribution is when all populations grow at the same rate, for instance, *N_i_*(*t*)=*N_i_*_0_ exp(*rt*), because in this case *P_i_*(*t*)=*N_i_*(*t*)/*J*(*t*) remains the same, and so does *θ* and *P_S_*. If one knows *P_S_* we can estimate the size of the original size of the population, *J’_S_*, as long as we know the relationship between *J*(*t*) and *J’_S_*. This, however, is only possible if the number of languages remains constant, a reasonable approximation if the probability of glossogenesis or the time interval between *J*(*t*) and *J’_S_* is small.
