# Supplementary material for: The relative abundance of languages: Neutral and non-neutral dynamics
Source: PLoS One. 2021 Dec 29;16(12):e0259162. doi: 10.1371/journal.pone.0259162 (PMC8716027; doi:10.1371/journal.pone.0259162)
Supplement: S3 Appendix — (DOCX) [file pone.0259162.s003.docx]

The relative abundance of languages:

neutral and non-neutral dynamics

Luís Borda-de-Água and Stephen P. Hubbell

**SUPPORTING INFORMATION**

**S3 The Random Fission and the Protracted Origination Modes**

Besides the peripheral isolation mode that leads to language abundance distributions described by the Allen Savage distribution, two other origination modes that are possible candidates to explain the observed language abundance distributions are the random fission and the protracted origination modes. However, as we show here, they are not appropriate to fit the observed LADs: The random fission does not lead to a good fit of the distributions and the protracted origination leads to parameters values that are not compatible with what we know about languages evolution.

Etienne and Haegeman (2011) developed the analytical expression for the random fission mode. Following the procedure described in the main text and using the same notation, the equivalent to equation (1) of the main text for random fission is

$$p(N)=\frac{\theta_{rf}^{2}}{J\sqrt{1-\frac{N}{J}}}\frac{I_{1}\left(2\theta_{rf}\sqrt{1-\frac{N}{J}}\right)}{I_{1}\left(2\theta_{rf}\right)}$$

where $\theta_{rf}=J\sqrt{\nu}$ and *I_n_*(*x*) is the modified Bessel function of the first kind for integer *n* and real-valued *x* (Abramowitz and Stegun 1981).

A typical example of the fitting provided by the maximum likelihood estimates from the above expression is shown in Fig. S.2 for the Indonesian LAD. As we can see the, the fitting (red) curve is clearly different from the empirical histogram.

**Figure S.2** **Typical example of the fitting provided by the random fission maximum likelihood estimates, red curve.** Notice the clear departure of the fitting distribution from the real data. The blue curve is the lognormal distribution fitted to the same data.

The protracted speciation mode was introduced by Rosindell et al. (2010). It assumes that a language arises from one single individual but as a gradual process so that a new language is only recognized after a certain number of generations after its incipiency: the two parameters to be estimated are the number of generations, *τ*, that has elapsed since origination until the new language is recognized as such, and the rate of origination-initiation, *μ*. The rate of origination-initiation, *μ*, is different from the rate of origination, *ν*, because the former is related to the origination of all languages, including those that do not survive *τ* generations until being finally recognized, while *ν* measures the rate of origination of only those languages that are identified. Therefore *ν* is smaller than *μ*, being related through

$\nu=\frac{\mu}{1+\tau}$,

and *ν*=*μ* only in the special case when *τ*=0.

Under the protracted origination mode, the equivalent probability expression to equation 1 of the main text is (Rosindell et al. 2010)

$$p(N)=\frac{1}{N}\left(\left(1-\frac{\mu}{1+\tau\mu}\right)^{N}-\left(1-\frac{1}{1+\tau}\right)^{N}\right)/\log\left(\frac{1+\tau\mu}{\mu+\tau\mu}\right)$$

We refer to this distribution as the “Rosindell et al” distribution.

We show in Fig. S3 some examples of the fitting provided by this distribution. As we can see, the fitting is almost identical to that provided by the Allen-Savage distribution, including the development of a plateau in some situations. However, the number of generations predicted often leads to the time to recognition of a new language, *T_rec_*, that is too long compared to what we know about language evolution. To show this we used the same growth rates and times to population size equilibrium as in the main text to estimate the population size at equilibrium periods, *J’*, and the time from the present to the last instant of equilibrium, Δ*T*. Because simulations showed that *τ* varies linearly with the total population size, *J*, we can then estimate *τ’* at population size equilibrium periods. If we assume that a typical generation time of 25 years (assuming shorter times do not significantly change the main conclusion), we obtain the *T_rec_* shown in Table S4 that are too large to be realistic.

**Fig. S.3** **This figure is equivalent to Fig. 1 of the main text, but in addition it shows the curves obtained with the protracted model distribution, green line in plots (a,d,g).** The bins are centered in integers numbers, *n*, and have borders at *n*±0.5. In the rank abundance distributions languages are ranked from the most abundant language on the left-hand side of the *x*-axis to the least abundant on the right-hand side. The errors bars correspond to 95% confidence intervals and were obtained for each country by sampling 200 times a number of points equal to the number of languages from a distribution with parameters corresponding to the maximum likelihood estimates. In order to distinguish the mode speciation modes, we call here the Allen-Savage distributions “peripheral isolation”, by analogy with the name used in ecological studies. Plots for Solomon Islands, Cameroon and Papua New Guinea.

**Fig. S.3** **This figure is equivalent to Fig. 1 of the main text, but in addition it shows the curves obtained with the protracted model distribution, green line in plots (j,m,p) (continuation).** Plots for Colombia, Indonesia and Philippines.

**Table S4.** **The estimated number of generations *τ’* and the time to recognition, *T_rec_*.** *T_rec_* was calculated assuming a generation time of 25 years.

| Country | *τ’*(generations)  *-*95%CI | *τ’*  (generations) | *τ’*(generations)  *+*95%CI | *T_rec_* (years)  -95%CI | *T_rec_* (years) | *T_rec_* (years)  +95%CI |
| --- | --- | --- | --- | --- | --- | --- |
| Malawi | 17000 | 44300 | 558000 | 33872.2 | 88267.1 | 1111806.5 |
| Zimbabwe | 9500 | 27700 | 138000 | 7769.7 | 22654.9 | 112865.5 |
| Niger | 2100 | 5100 | 21800 | 4560.2 | 11074.8 | 47339.4 |
| Namibia | 1250 | 3200 | 18350 | 4269.4 | 10929.7 | 62675.2 |
| Guinea | 1100 | 2600 | 9950 | 1073.0 | 2536.2 | 9705.8 |
| Liberia | 16000 | 24300 | 114500 | 9148.8 | 13894.7 | 65470.8 |
| Zambia | 6800 | 16500 | 60900 | 13548.9 | 32876.0 | 121342.3 |
| Gabon | 1650 | 2800 | 16250 | 3583.0 | 6080.3 | 35287.4 |
| Angola | 1800 | 4700 | 17100 | 7087.1 | 18505.1 | 67327.0 |
| Togo | 3000 | 7600 | 42500 | 1311.4 | 3322.2 | 18578.0 |
| Uganda | 1800 | 54500 | 16900 | 1116.1 | 33794.3 | 10479.3 |
| Mozambique | 8200 | 18400 | 122600 | 10950.4 | 24571.7 | 163722.0 |
| Mali | 3100 | 7200 | 30700 | 6731.8 | 15635.0 | 66666.1 |
| Congo | 1400 | 3010 | 7000 | 3040.1 | 6536.3 | 15200.7 |
| Burkina Faso | 1200 | 2950 | 8600 | 1035.5 | 2545.7 | 7421.4 |
| Cent. Afr. Republic | 2200 | 4400 | 14000 | 4777.4 | 9554.8 | 30401.5 |
| Ghana | 1600 | 3580 | 8600 | 169.4 | 379.0 | 910.5 |
| Côte d’Ivoire | 1800 | 4010 | 11000 | 357.3 | 796.0 | 2183.5 |
| Tanzania | 10200 | 18100 | 39200 | 6883.6 | 12215.0 | 26454.6 |
| Chad | 560 | 1010 | 2240 | 1216.1 | 2193.2 | 4864.2 |
| Dem. Rep. Congo | 2780 | 4140 | 6600 | 3712.5 | 5528.6 | 8813.7 |
| Cameroon | 920 | 1370 | 2140 | 1997.8 | 2975.0 | 4647.1 |
| Guyana | 5.4 | 14.7 | 5.41E+02 | 11.7 | 31.9 | 1174.4 |
| Panama | 6 | 8.8 | 7.46E+02 | 1.5 | 2.2 | 182.7 |
| Suriname | 10 | 21 | 7.21E+02 | 21.7 | 45.6 | 1565.7 |
| Oman | 24 | 203 | 164 | 238.1 | 2014.0 | 1627.1 |
| Laos | 301 | 670 | 1501 | 957.3 | 2130.9 | 4773.9 |
| Solomon Islands | 700 | 1290 | 5200 | 4622.0 | 8517.7 | 34334.9 |
| Vanuatu | 90 | 151 | 290 | 594.3 | 997.0 | 1914.8 |
| Papua New Guinea | 247 | 301 | 373 | 1630.9 | 1987.5 | 2462.9 |

**References**

Abramowitz, M., Stegun, I.A. eds., 1964. Handbook of mathematical functions with formulas, graphs, and mathematical tables (Vol. 55). US Government printing office.

Etienne, R. S., Haegeman, B., 2011. The neutral theory of biodiversity with random fission speciation. Theoretical Ecology 4, pp. 87-109. https://doi.org/10.1007/s12080-010-0076-y

Rosindell, J., Cornell, S.J., Hubbell, S.P. and Etienne, R.S., 2010. Protracted speciation revitalizes the neutral theory of biodiversity. Ecology Letters 13, pp.716-727. <https://doi.org/10.1111/j.1461-0248.2010.01463.x>
