# Supplementary material for: The relative abundance of languages: Neutral and non-neutral dynamics
Source: PLoS One. 2021 Dec 29;16(12):e0259162. doi: 10.1371/journal.pone.0259162 (PMC8716027; doi:10.1371/journal.pone.0259162)
Supplement: S4 Appendix — (DOCX) [file pone.0259162.s004.docx]

The relative abundance of languages:

neutral and non-neutral dynamics

Luís Borda-de-Água and Stephen P. Hubbell

**SUPPORTING INFORMATION**

**S4 Statistics for *θ* and the predicted origination rate *ν’* assuming a past period of total population size in equilibrium.**

The purpose of this appendix is mainly to show the potential of apply our approach to obtain a better understanding of the historical dynamics of language origination. For those countries with a good fit according to the 95% confidence intervals in the rank plots (Fig. S1), mostly the African and Oceania countries, we can make the neutral (non-differential) growth assumption and use *θ* and *P_S_* to estimate the population size, *J’_S_*=*P_S_***J’*, and the origination rate, *ν’*=*θ* /2*J’*, at the last point in time when the assumption of a total population size, *J’*, being constant is reasonable. To estimate *J’* we used data from McEvedy and Jones (1978), but further research in this area requires better estimates. The estimated values of *J’_S_* and *ν’* are in Table S5, together with the interval of time assumed between the past period of equilibrium and the present, Δ*T*.

Using only the African countries because they form the geographical and historical unit where more LADs were well fitted by the Allen-Savage distribution, we observe from Fig. S.4a and S.4b that the number of languages varies linearly with *θ* but that it has no correlation with the origination rate *ν’*. This result was expected because according to neutral theory the total number of languages is determined not only by the origination rate but also by the total number of individuals, the two parameters that enter into the definition of *θ*.

Incidentally, our results give credit to Nettle’s (1999) hypothesis on the relationship between language richness and ecological risk, the latter being inversely proportional to the mean growing season. According to Nettle, when the growing season increases the interdependence among different populations decreases, favoring the divergence of languages and, hence, the increase in their richness. Therefore, we expect larger language origination rates in regions with larger mean growing seasons, and that is what we observe in Fig. S4c.

Using all countries in Table S5, we see that the origination rate, *ν’*, exhibits a large range of variation, approximately three orders of magnitude. The three Oceania countries have the largest origination rates; the largest being that of Vanuatu, the second that of Solomon Islands and the third of Papua New Guinea. Given the small number of Asian and American countries it is difficult to draw generalizations about their *ν’*. For Africa, the six countries with the largest *ν’* are in the Gulf of Guinea region, Fig. S5a, which are, by decreasing value of *ν’*: Gabon, Côte d’Ivoire, Liberia, Ghana, Togo and Cameroon. Interestingly, the region with the highest *ν’* is also that where the Niger-Congo (including the Bantu) languages arose (e.g., Diamond 1997).

**Fig. S.4. Statistics for *θ* and the predicted origination rate *ν’* for a period of equilibrium population size.** Plots a and b show the relationship between the number of languages and *θ* and *ν’*, respectively. Plot d shows the positive relationship between *ν’* and the mean growing season.

**Fig. S.5.** **Maps of Africa showing in shades of gray (a) the origination rates, *ν’*, and (b) the population sizes at the origination of a language, *J’_S_*.** Notice that the countries with the largest *ν’* and the smallest *J’_S_* are predominantly in the west coast of Africa in the region of the Gulf of Guinea.

**Table S5. Predicted population size at origination, *J’_S_*, and the rate of origination, *ν’*, for the countries with observed good fitting, as revealed by the rank abundance plots** **(see Fig. S1 in Supporting Information).** These values were based on estimates of the population growth rate, *r*, and time to population size equilibrium, Δ*T* (McEvedy and Jones 1978). The mean growing season (mgs) values were taken from Nettle (1999).

| **Continent** | **Country** | **r (year^-1^)** | **Δ*T* (years)** | ***J’_S_*** | ***ν’*** | **mgs** |
| --- | --- | --- | --- | --- | --- | --- |
| Africa | Malawi | 0.025 | 100 | 820782 | 1.46E-06 | 5.80 |
| Africa | Zimbabwe | 0.034 | 100 | 516128 | 2.52E-06 | 5.29 |
| Africa | Niger | 0.012 | 200 | 801805 | 2.12E-06 | 2.40 |
| Africa | Namibia | 0.020 | 100 | 254486 | 9.04E-06 | 2.50 |
| Africa | Guinea | 0.016 | 200 | 259102 | 8.10E-06 | 7.38 |
| Africa | Liberia | 0.019 | 200 | 55579 | 9.18E-05 | 7.14 |
| Africa | Zambia | 0.025 | 100 | 850652 | 4.64E-06 | 5.43 |
| Africa | Gabon | 0.012 | 200 | 52935 | 1.12E-04 | 8.79 |
| Africa | Angola | 0.009 | 200 | 1728991 | 1.68E-06 | 6.22 |
| Africa | Togo | 0.020 | 200 | 69562 | 5.75E-05 | 7.91 |
| Africa | Uganda | 0.018 | 200 | 421760 | 1.30E-05 | 10.14 |
| Africa | Mozambique | 0.015 | 200 | 865895 | 4.68E-06 | 6.07 |
| Africa | Mali | 0.012 | 200 | 782705 | 5.69E-06 | 3.59 |
| Africa | Congo | 0.012 | 200 | 292385 | 2.05E-05 | 9.60 |
| Africa | Burkina Faso | 0.017 | 200 | 380343 | 1.31E-05 | 5.17 |
| Africa | Cent. Afr. Republic | 0.012 | 200 | 286533 | 2.79E-05 | 8.08 |
| Africa | Ghana | 0.027 | 200 | 85225 | 6.45E-05 | 8.79 |
| Africa | Côte d'Ivoire | 0.024 | 200 | 72654 | 1.03E-04 | 8.67 |
| Africa | Tanzania | 0.018 | 200 | 693425 | 2.02E-05 | 7.02 |
| Africa | Chad | 0.012 | 200 | 504612 | 2.08E-05 | 4.00 |
| Africa | Dem. Rep. Congo | 0.015 | 200 | 2051173 | 8.53E-06 | 9.44 |
| Africa | Cameroon | 0.012 | 200 | 837096 | 3.34E-05 | 9.17 |
| America | Guyana | 0.012 | 200 | 58796 | 7.65E-06 | 12.00 |
| America | Panama | 0.023 | 200 | 20575 | 1.94E-05 | 9.20 |
| America | Suriname | 0.012 | 200 | 54620 | 1.28E-05 | 12.00 |
| Asia | Oman | 0.009 | 100 | 583394 | 1.11E-06 | 0.00 |
| Asia | Laos | 0.010 | 200 | 674288 | 8.90E-06 | 7.14 |
| Oceania | Solomon Islands | 0.013 | 100 | 93494 | 1.55E-04 | 12.00 |
| Oceania | Vanuatu | 0.013 | 100 | 31031 | 4.83E-04 | 12.00 |
| Oceania | Papua New Guinea | 0.013 | 100 | 968081 | 9.97E-05 | 10.88 |

**References**

Abramowitz, M., Stegun, I.A. eds., 1964. Handbook of mathematical functions with formulas, graphs, and mathematical tables (Vol. 55). US Government printing office.

Diamond, J., 1997. Guns, germs and steel: a short history of everybody for the last 13,000 years. W. W. Norton & Company, New York.

McEvedy, C., Jones, R., 1978, Atlas of the World Population History. Peguin Books, Middlesex, England.
